# Effect of a Novel Engagement Strategy Using Twitter on Test Performance

**DOI:** 10.5811/westjem.2015.10.28869

**Published:** 2015-11-12

**Authors:** Amanda L. Webb, Adam Dugan, Woodrow Burchett, Kelly Barnett, Nishi Patel, Scott Morehead, Mark Silverberg, Christopher Doty, Brian Adkins, Lauren Falvo

**Affiliations:** * University of Kentucky College of Medicine, Lexington, Kentucky; † University of Kentucky, Department of Statistics, Lexington, Kentucky; ‡ University of Kentucky, Department of Emergency Medicine, Lexington, Kentucky

## Abstract

**Introduction:**

Medical educators in recent years have been using social media for more penetrance to technologically-savvy learners. The utility of using Twitter for curriculum content delivery has not been studied. We sought to determine if participation in a social media-based educational supplement would improve student performance on a test of clinical images at the end of the semester.

**Methods:**

116 second-year medical students were enrolled in a lecture-based clinical medicine course, in which images of common clinical exam findings were presented. An additional, optional assessment was performed on Twitter. Each week, a clinical presentation and physical exam image (not covered in course lectures) were distributed via Twitter, and students were invited to guess the exam finding or diagnosis. After the completion of the course, students were asked to participate in a slideshow “quiz” with 24 clinical images, half from lecture and half from Twitter.

**Results:**

We conducted a one-way analysis of variance to determine the effect Twitter participation had on total, Twitter-only, and lecture-only scores. Twitter participation data was collected from the end-of-course survey and was defined as submitting answers to the Twitter-only questions “all or most of the time”, “about half of the time”, and “little or none of the time.” We found a significant difference in overall scores (p<0.001) and in Twitter-only scores (p<0.001). There was not enough evidence to conclude a significant difference in lecture-only scores (p=0.124). Students who submitted answers to Twitter “all or most of the time” or “about half the time” had significantly higher overall scores and Twitter-only scores (p<0.001 and p<0.001, respectively) than those students who only submitted answers “little or none of the time.”

**Conclusion:**

While students retained less information from Twitter than from traditional classroom lecture, some retention was noted. Future research on social media in medical education would benefit from clear control and experimental groups in settings where quantitative use of social media could be measured. Ultimately, it is unlikely for social media to replace lecture in medical curriculum; however, there is a reasonable role for social media as an adjunct to traditional medical education.

## INTRODUCTION

The medical profession is one steeped in tradition. As many pre-medical hopefuls are informed – medicine is not only a career; it is a lifestyle dedicated to constant inquiry and the ascertainment of knowledge. Physicians are expected to grow and adapt to evolutions in their patients’ environments. So, too, must the medical curriculum adjust to the millennial generation’s affinity for technologically-driven mediums. In a 2011 study, Bosslet et al found that personal online social network (OSN) use among physicians and physicians-in-training was comparable to the general population; specifically, 90+% of medical students endorsed some form of OSN use,^1^ with Facebook recognized as the most popular site on the Internet,^2^ and Twitter reporting 302 million active users as of April 2015.^3^ The advent of the social media age calls for a rapid adjustment in communication methods and curriculum goals. A 2013 systematic review by Cheston et al explored 14 studies’ reports on the effects of social media in medical education. In their discussion, they stated that the use of social media in medical education merited further exploration, and suggested the benefit of more studies with “clear definitions of social media technologies… to allow appropriate comparisons and data synthesis.^4^

With these study deficits in mind, we sought to explore the use of a single social media focus (Twitter) and its effect on information exposure and retention within a second-year medical school class.

## METHODS

During the 2013–2014 academic year, 116 second-year medical students were mandatorily enrolled in Introduction to Clinical Medicine II (ICM2) as part of their curriculum. All students enrolled in this course were considered responsible for its lecture content, per the syllabus distributed and reviewed on the first day of class. Within the course’s weekly lecture series, images of clinically-relevant physical exam findings were presented to students. Outside the requirements of the class, students were invited to create a Twitter account and participate in an optional “Twitter Question of the Week.” In this assessment, a short clinical scenario, accompanied by imaging of a relevant physical finding, was “tweeted out” weekly at pre-determined and publically-announced intervals. The twitter-associated clinical scenario and image were selected from content not covered in the ICM2 mandatory curriculum. Students were challenged to name the diagnosis based off the presented data. Student answers were submitted by direct messaging to the course instructor’s account. The time stamp from each student’s message enabled the instructor to identify the first 20 correct answers each week. These first 20 correct respondents were awarded “bonus points” (worth a fraction of a percent) to be added to their next ICM2 exam grade. Over the course of the year, over 80 students attempted an answer in at least one “Twitter Question of the Week.”

After the completion of the course, students were asked to participate in a slideshow “quiz,” with no effect on their grade. One hundred sixteen students (100% participation) were tested over their recognition of 24 clinical images—12 images from the lecture hours, and 12 images from the weekly twitter questions. These 24 images were assessed by a physician and third-year clinical medical student and selected for their straightforward, commonplace physical exam findings. Each image was projected for 30 seconds while students used pencil and paper to name the associated diagnosis or relevant exam finding. Course-based and Twitter-based photos were alternated in a 1:1 fashion.

On the same paper as their “quiz” answers, students were asked to complete a seven-point survey regarding each student’s use of Twitter prior to the ICM course, his/her level of participation in the “Twitter Question of the Week” (did the student follow the ICM2 account? How many times did a student see/submit an answer to the twitter account? How many times did the student earn a “top 20” response?), and his/her reflections on educational Twitter usage (did the student feel like s/he retained more information regarding physical exam findings? Did this account enhance the student’s overall education in ICM2?). Quiz/survey answers were then collected and analyzed by a statistician within the university.

## RESULTS

**Figure 1:**
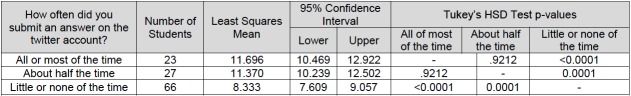
Least-squares means for all questions by answering questions on Twitter. *HSD,* honest significant difference

**Figure 2:**
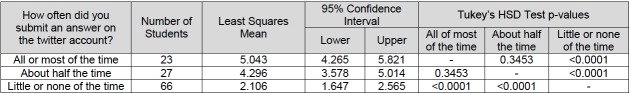
Least-squares means for Twitter exclusive questions by answering questions on Twitter. *HSD*, honest significant difference

A one-way analysis of variance (ANOVA) was conducted to determine the effect Twitter participation had on total scores, Twitter-only scores, and lecture-only scores. Twitter participation data was collected from the end-of-course survey and was defined as submitting answers to the Twitter-only questions “all or most of the time”, “about half of the time”, and “little or none of the time.” The one-way ANOVA (Figure 1) determined that there was a significant difference in overall scores (p<0.001) and in Twitter-only scores (p<0.001), but there was not enough evidence to conclude a significant difference in lecture-only scores (p=0.124). A post hoc analysis using Tukey’s range test (Figure 2) determined that those students who submitted answers to Twitter “all or most of the time” or “about half the time” had significantly higher overall scores (p<0.001 and p<0.001, respectively) and significantly higher Twitter-only scores (p<0.001 and p<0.001, respectively) than those students who only submitted answers “little or none of the time.”

## DISCUSSION

As the integration of social media into undergraduate and continuing medical education is still in its infancy, much of the literature has focused on descriptive terminology and anecdotal case studies. Generally, social medial integration into the curriculum has been well received by students and increases engagement, collaboration, and feedback, which may explain current trends to incorporate social media into medical education.^5^–^9^ However, no substantial evidence exists to suggest that social media platforms are a comparable or superior form of medical education compared to current curriculums.^10^–^12^ In light of these studies, as well as the results of our own research, we submit that while Twitter is not an appropriate replacement for aspects of medical education, it may have a role as an adjunct to traditional curriculums, as well as serve as another outlet to engage medical students on a more individual level. Within our research, students scored, on average, 54% of the course content questions correctly, and 27% of the Twitter questions correctly. Although their level of retention was not as high when compared to traditional classroom lectures, students who participated in the “Twitter Question of the Week” did retain some information from that medium, and a majority of the students in the class felt that Twitter added to their education.

This research study has several limitations. By declaring involvement in the Twitter project optional, the study risks self-selecting students who are already more academically-engaged into the Twitter-participant group. Alternatively, because the Twitter quizzes did not count towards ICM grades, students may have been less likely to return to and review the material outside of that week’s presentation. Additionally, the slideshow quiz at the end of the course used the exact photos from Twitter questions, allowing for the possibility that students simply recognized the photo at hand, rather than interpreted the findings and demonstrated understanding of the medical condition.

In light of these limitations, future research on social media’s role in medical education would benefit from clear control and experimental groups in settings where quantitative use of social media could be measured. If this study were to be replicated at other sites, it would be beneficial to use other photos/presentations of the selected exam findings to more accurately measure students’ comprehension of the diseases. Ultimately, it is unlikely for social media to replace lecture within the medical curriculum; however, there is a reasonable role for social media as an adjunct to traditional medical education.
